# Dactylogyridae (Monopisthocotyla) parasitizing *Caenotropus labyrinthicus* (Characiformes: Chilodontidae) from the Tocantins and Gurupi river basins, northern Brazil, with the proposal of *Tocantinsiella* n. gen. (Dactylogyridae) and four new species

**DOI:** 10.1007/s11230-026-10303-w

**Published:** 2026-10-30

**Authors:** Jéssica França Antunes, Marcia Cristina Nascimento Justo, Yuri Costa de Meneses, Álvaro José Bittencourt de Freitas, Diego Carvalho Viana, Felipe Polivanov Ottoni, Simone Chinicz Cohen

**Affiliations:** 1 Laboratório de Helmintos Parasitos de Peixes – LHPP, Instituto Oswaldo Cruz, Fiocruz, Avenida Brasil, 4365, Manguinhos, Rio de Janeiro, RJ 21040‐360 Brazil; 2 Núcleo de Estudos Morfofisiológicos Avançados – NEMO, Universidade Estadual da Região Tocantina do Maranhão, Campus Imperatriz, Rua Godofredo Viana, 1300, Imperatriz, MA 65901‐480 Brazil; 3 Laboratório de Sistemática e Ecologia de Organismos Aquáticos, Universidade Federal do Maranhão (UFMA), Campus de Chapadinha, BR-222, KM 04, S/N, Chapadinha, MA Brazil

## Abstract

Four new species, assigned to the proposed new genus *Tocantinsiella* **n. gen.,** were described from the gills of *Caenotropus labyrinthicus* collected in the Tocantins and Gurupi river basins, northern Brazil: *Tocantinsiella maranhensis* **n. sp.**, *Tocantinsiella brassicae* **n. sp.**, *Tocantinsiella vesiculosa* **n. sp.**, and *Tocantinsiella flabellata* **n. sp.** The new species are characterized mainly by the presence of a sclerotized, robust, and sinistral vaginal vestibule, as well as a copulatory ligament connecting the male copulatory organ (MCO) from the accessory piece, which represent morphological and diagnostic characteristics for the proposed new genus. *Tocantinsiella* **n. gen.** is morphologically similar to *Heterothecium* due to the presence of a sclerotized, sinistral vaginal vestibule, differing due to the presence of a copulatory ligament and a non-articulated MCO. *Philocorydoras* also shares similarities related to the morphology of the copulatory complex. However, species of this genus are recorded only in Siluriformes fishes. This study provides a significant expansion of the diversity of helminth fauna in fishes of the family Chilodontidae by describing a new genus and four new species of Monopisthocotyla in the host studied.

## Introduction

The Neotropical Region is home to a rich diversity of freshwater fishes, accounting for 20% to 25% of global diversity. The Tocantins River basin, the largest river system located entirely within the Brazilian territory, is one of the rivers with the highest number of endemic taxa, including a great diversity of fish species (Cohen et al., 2020; Malabarba & Malabarba, 2020; Chamon et al., 2022). The Gurupi River basin, in turn, is an important river system located along the border between the states of Maranhão and Pará, Brazil (Koerber et al., 2022). However, the pressure exerted by anthropogenic threats directly impacts the maintenance of fauna biodiversity in Brazilian freshwater systems (Albert et al., 2021; Ottoni et al., 2023, 2025).

Chilodontidae is a family of freshwater fishes with few described species when compared to other taxa within the order Characiformes. However, they are widely distributed in Brazilian river basins (Torrente-Vilara et al., 2013). To date, only two genera belonging to the family Chilodontidae are known: *Chilodus* Müller & Troschel and *Caenotropus* Günther, which have eight valid species. Among them, stands out *Caenotropus labyrinthicus* (Kner), popularly known as “joão-duro”. This species is distributed in the main basins of South America and is characterized by its small size in relation to other Characiformes (Vari et al., 1995).

In terms of parasitic ichthyofauna, Monopisthocotyla is one of the most diverse groups, mainly by its different morphology and colonization in several host species. Monopisthocotylan parasites on freshwater fishes are most represented by members of the family Dactylogyridae Bychowsky (Cohen et al., 2013; Tavares-Dias et al., 2022). However, there are only four records of parasitism by Monopisthocotyla in chilodontids, which is the proposition of two new species of *Urocleidoides* Mizelle & Price, 1964 and new data on *Urocleidoides tocantinensis* Freitas, Bezerra, Meneses, Justo, Viana & Cohen, 2021 and on *Anacanthorus simpliciphallus* Silva, Cohen, Costa & Justo, 2024 in *C. labyrinthicus* (Freitas et al., 2021, 2025; Bezerra et al., 2023).

During studies on the helminth fauna of fishes from the Tocantins River, four new species of *Tocantinsiella* **n. gen.** were found parasitizing the gill filaments of *C. labyrinthicus* and described based on their morphological and morphometric data. The results contribute to expanding knowledge about the parasitic diversity present in fish in the region, specifically in the family Chilodontidae.

## Materials and methods

During October and November 2023 and November and December 2024, 71 specimens of *C. labyrinthicus* were collected in the Tocantins River, state of Maranhão, near the urban area of Imperatriz, in the village of Embiral (5°27'50’’S, 47°33'48’’W), which belongs to the rural area of the same municipality. Fish specimens were caught using nets, with the aid of local fishermen, stored in refrigerated boxes containing ice, and transported to the “Universidade Estadual da Região Tocantina do Maranhão” (UEMASUL), Maranhão State, Brazil, where they were identified and necropsied for parasitological analysis. At the “Laboratório de Ecologia e Limnologia”, Universidade Estadual da Região Tocantina do Maranhão” (UEMASUL), gill filaments of each specimen were removed and placed in vials containing hot water at approximately 65 °C, followed by shaking of the vials to detach the parasites from the gills. After this process, absolute ethanol was added to reach a concentration of 70%. During June 2025, eight specimens of *C. labyrinthicus* were collected in the Piriá River, Gurupi River basin, Beiradão village, municipality of Paragominas (3°20'06.0’’S, 46°52'24.8’’W) Pará State, Brazil (at night, using a cast net and a 3-m-long seine net). In the collecting site, the gill filaments of each specimen were removed and placed in vials containing hot water, at approximately 65 °C, followed by shaking of the vials to detach the parasites from the gills. After this process, absolute ethanol was added to reach a concentration of 70%. The previously fixed samples were properly preserved and sent to the “Laboratório de Helmintos Parasitos de Peixes, Instituto Oswaldo Cruz”, Fiocruz, Rio de Janeiro state, Brazil, where the gills and sediments were observed under a stereoscopic microscope for monopisthocotylan species collection. Monopisthocotylans were mounted in Hoyer's medium for studies of the sclerotized parts. The measurements, presented in micrometers, were taken using the micrometric eyepiece on a Zeiss Axioskop® 2 light microscope (Jena, Germany), with the averages represented in parentheses, followed by the number of measurements taken (Table 1). Illustrations of the parasites were also made using a drawing tube attached to a Zeiss Axioskop^®^ 2 light microscope and were subsequently photographed with a digital camera. The identification of authors and nomenclatural acts for taxon followed the guidelines mentioned in Article 50.1 and recommendation 50A of the International Code of Zoological Nomenclature (ICZN, 1999), which refers specifically to the identity of authorship. Quantitative descriptors of the parasitic populations follow Bush et al. (1997). The representative specimens studied were deposited in the “Coleção Helmintológica do Instituto Oswaldo Cruz (CHIOC)”, from “Fundação Oswaldo Cruz, Fiocruz” in the state of Rio de Janeiro, Brazil.

**Table 1:** Comparative measurements (in micrometers) of the four new species of *Tocantinsiella* **n. gen.***,* parasites of *Caenotropus labyrinthicus* from the Tocantins River, in the state of Maranhão

Measurements	*Tocantinsiella maranhensis* **n. sp.**	*Tocantinsiella brassicae* **n. sp.**	*Tocantinsiella vesiculosa*** n. sp.**	*Tocantinsiella flabellata ***n. sp.**
Body width	109 (105–113)	84 (55–138)	87 (34–123)	76 (68–83)
Body length	344 (343–345)	281 (170–375)	228 (160–295)	288 (255–320)
Haptor width	56 (50–60)	58 (45–90)	65 (23–88)	48 (30–58)
Pharynx	23	24 (21–28)	15	23 (16–30)
MCO	33 (22–42)	25 (16–36)	32 (27–35)	23 (20–30)
Accessory piece	31 (30–34)	28 (15–36)	29 (28–32)	21 (16–28)
Vagina width	11 (10–12)	9 (5–12)	14 (10–18)	10 (6–13)
Vagina length	20 (18–21)	16 (10–25)	22 (15–30)	13 (10–15)
Ventral anchor width	14 (13–15)	11 (7–16)	14 (11–16)	13 (11–16)
Ventral anchor length	27 (20–30)	18 (12–26)	20 (15–25)	18 (12–23)
Ventral bar length	21 (16–24)	10 (8–11)	35	15 (14–16)
Dorsal anchor width	14 (12–15)	9 (6–15)	12 (10–14)	7 (3–11)
Dorsal anchor length	23 (20–30)	22 (12–31)	22 (15–28)	19 (17–20)
Dorsal bar length	23 (19–25)	18 (12–25)	26 (25–27)	15 (8–18)
Hooks	13 (12–14)	13 (11–15)	20 (15–23)	13 (11–14)
Egg diameter	–	50	–	–

## Results

TAXONOMY

Class Monopisthocotyla Brabec, Salomaki, Kolísko, Scholz & Kuchta, 2023

Subclass Polyonchoinea Bychowsky, 1937

Order Dactylogyridea Bychowsky, 1937

Dactylogyridae Bychowsky, 1933

***Tocantinsiella*** **n. gen.**

Type-species: *Tocantinsiella maranhensis* **n. sp.**

Other species: *Tocantinsiella brassicae* **n. sp.**, *Tocantinsiella vesiculosa* **n. sp.**, *Tocantinsiella flabellata* **n. sp.**

Etymology: The name of the genus refers to the Tocantins River where the host species was collected. The suffix *-ella* is a Latin diminutive appended to the genus name, which should be treated as feminine.

**Diagnosis.** Body elongated, fusiform, divided into cephalic region, trunk, peduncle, and haptor. Tegument thin, smooth. Cephalic lobes developed or not; three pairs of bilateral head organs. Cephalic glands not observed. Two pairs of eyespots; accessory granules present or absent. Mouth subterminal, midventral; pharynx muscular, glandular; esophagus short; two intestinal ceca, confluent posterior to testis, lacking diverticula. Gonads intercaecal, overlapping; testis dorsal to germarium. Vas deferens looping left intestinal cecum; seminal vesicle sigmoid, a dilation of vas deferens; prostatic reservoir single. Genital pore midventral near level of cecal bifurcation. Copulatory complex comprising male copulatory organ (MCO) and accessory piece; MCO consisting of a sclerotized tube with an ornate base or not; presence of a copulatory ligament extending from the accessory piece and linked to the base of the MCO. Accessory piece elongated and sclerotized. Vagina slightly sclerotized; vaginal aperture sinistral; vaginal vestibule sclerotized and robust. Seminal receptacle small. Haptor subhexagonal or subspherical, with pairs of dorsal and ventral anchors and bars; anchors with more sclerotized regions or not; 7 pairs of hooks, 5 pairs ventral, 2 pairs dorsal, similar in length or not with ancyrocephalinae distribution; hooks divided in two subunits or not. Gill parasites of chilodontid fishes.

**Remarks**

*Tocantinsiella* **n. gen.** is proposed to accommodate dactylogyrids that present the following characteristics combined: (1) two pairs of eyespots; (2) sclerotized and robust vaginal vestibule, in the sinistral position; (3) MCO composed of a long and sclerotized tube; (4) presence of a copulatory ligament that connects the accessory piece to the base of the MCO; (5) hooks of similar or different sizes, with or without subunits, in opposite positions or not. *Tocantinsiella* **n. gen.** exhibits characteristics like those of *Heterothecium* Kritsky, Boeger & Jégu, 1997 originally described in serrasalmids, mainly due to the morphology of the vaginal vestibule, in which both genera possess a sclerotized vaginal vestibule on the left side. Despite this similar characteristic, the new genus can be easily distinguished by the morphology of the copulatory complex, as species of *Heterothecium* possess a copulatory complex divided into two branches, differing from *Tocantinsiella* **n. gen.** which has a copulatory complex containing only one branch, in addition to the presence of a copulatory ligament. Furthermore, the new genus is characterized by the presence of a single prostatic reservoir, in contrast to species of *Heterothecium*, which possesses two reservoirs. *Tocantinsiella* **n. gen.** is also similar to *Philocorydoras* Suriano, 1986 regarding the morphology of the copulatory complex, but differs in the vaginal morphology and the presence of a single pair of eyespots, characteristics not observed in the species of *Tocantinsiella* **n. gen.** Furthermore, *Philocorydoras* species have been described in fishes of the order Siluriformes. Although there are similar morphological characteristics among *Heterothecium*, *Philocorydoras*, and *Tocantinsiella* **n. gen.**, there are significant differences that support the proposal of *Tocantinsiella* as a new genus.

**Fig. 1:**
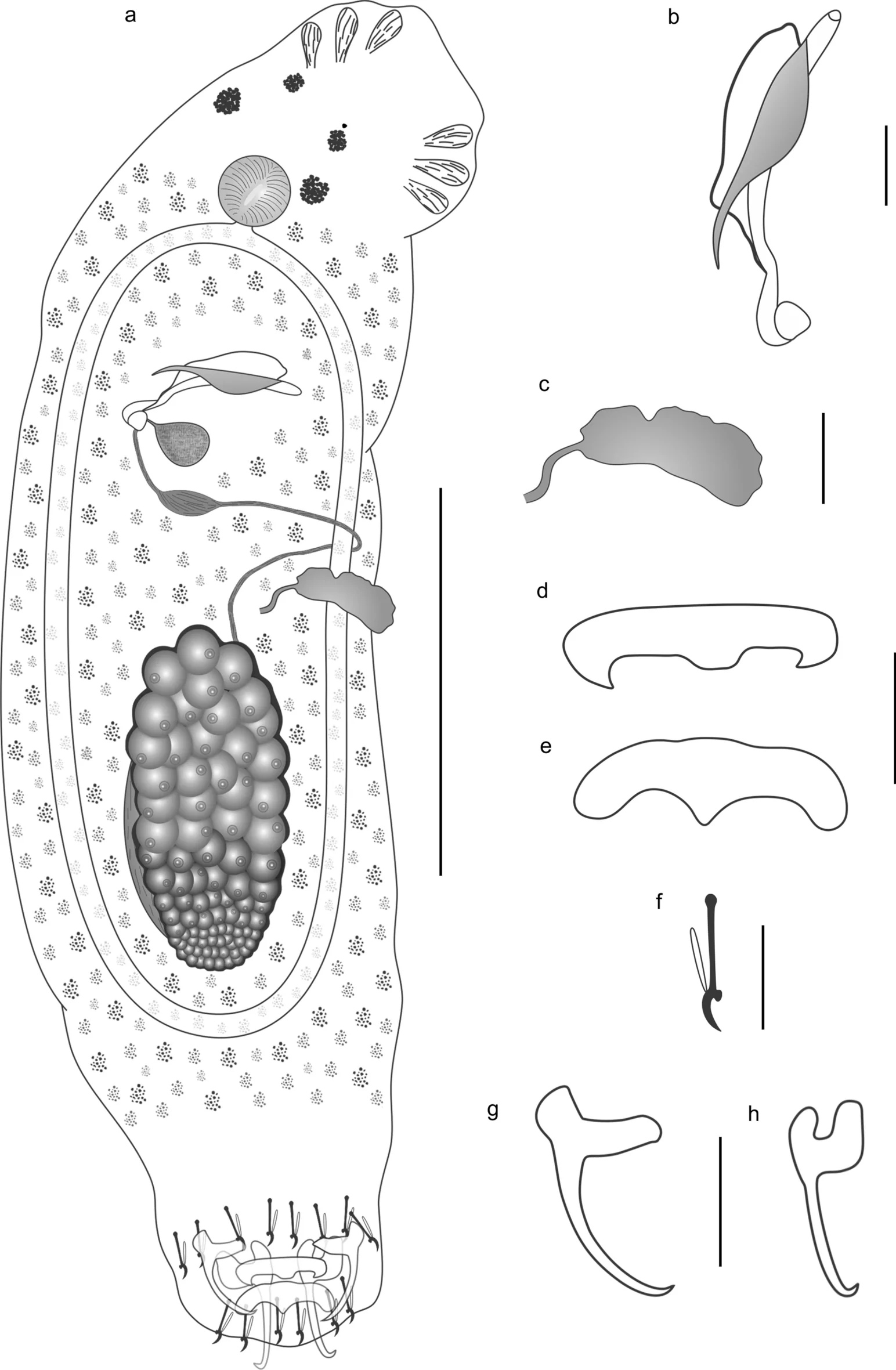
*Tocantinsiella maranhensis* **n. sp.** parasite of *Caenotropus labyrinthicus* from the Tocantins River, Maranhão State, Brazil. a, Total view, ventral (composite); b, Copulatory complex, ventral view; c, Vagina, ventral view; d, Ventral bar; e, Dorsal bar; f, Hook; g, Ventral anchor; h, Dorsal anchor. Scale bars: (a) 100 μm; (b–f) 10 μm; (g–h) 15 μm

***Tocantinsiella maranhensis*** **n. sp.** Antunes, Justo, Meneses, Freitas & Cohen (Fig. 1a–h)

Type-host: *Caenotropus labyrinthicus* (Kner) (Characiformes: Chilodontidae)

Type-locality: Tocantins River, near the urban area of Imperatriz, in the village of Embiral (5°27′50″S, 47°33′48″W). Maranhão State, Brazil.

Type-material: Holotype CHIOC: 41138; Paratypes CHIOC: 41139, 41140

Site of infestation: Gills.

Parasitological indices: Prevalence 5.6% (4/71 infested); Mean abundance of infestation: 0.05; Mean intensity of infestation: 1; Amplitude of intensity: 1.

Etymology: The specific name refers to the location (Maranhão State, Brazil) where the host species was collected.

### Description (Fig. 1a–h)

(Based on 4 specimens mounted in Hoyer’s medium): Body fusiform 343 and 345 (n = 2) long, 105 and 113 (n = 2) wide. Tegument smooth. Cephalic lobes moderately developed, with three bilateral pairs of head organs. Two pairs of eyespots present, anterior pair smaller and closer than posterior pair. Accessory eye granules not observed. Pharynx spherical 23 (n = 1). Two intestinal caeca confluent posterior to gonads. Germarium elongated and semioval-shaped, 60 (n = 1) long and 35 (n = 1) wide. Mehlis’ gland, oviduct, ootype, uterus, and egg not observed. Vaginal opening marginal, sinistral, near the midlength of the body. Vagina robust and slightly sclerotized, 20 (18–21; n = 3) long, 11 (10–12; n = 3) wide. Vitellaria present throughout the trunk, surrounding the reproductive organs. Testis dorsal to germarium. Vas deferens looping left intestinal cecum. Seminal vesicle a dilation of vas deferens, elongated, oval-shaped. Single prostatic reservoir saccate. Male copulatory organ (MCO) comprises a tube slightly sclerotized, 33 (22–42; n = 4) long. Accessory piece sclerotized, rod-shaped, widening towards the distal portion, 31 (30–34; n = 4) long. Copulatory ligament connecting the base of the MCO to the accessory piece. Haptor subspherical, 56 (50–60; n = 4) wide. Ventral bar anteriorly straight, ends tapered and turned inwards with a short, more enlarged process in the median region of the posterior part, 21 (16–24; n = 3) wide. Dorsal bar with ends turned downwards and a pointed process in the posterior median region 23 (19–25; n = 4) wide. Ventral anchor delicate with a deep root elongated and slightly pointed at the end, superficial root elongated and arranged laterally, slightly rounded, straight and long shaft and curved point 27 (20–30; n = 4) long, 14 (13–15; n = 4) wide. Dorsal anchor robust, deep root with rounded end, superficial root dilated and rounded, projecting upwards, parallel to deep root, straight shaft, short point, 23 (20–30; n = 4) long and 14 (12–15; n = 4) wide. Seven pairs of hooks displayed upside down, with the base facing upward, similar in size and shape with straight shaft, protruded thumb, curved point, shank with bulbous base, 13 (12–14; n = 4) long. Filamentous hooklet (FH) loop about 2/3 of the total length.

Remarks: *Tocantinsiella maranhensis* **n. sp.** is distinguished by the morphology of dorsal bar, which is characterized by the presence of posteromedial protuberance, and a ventral bar with ends tapered and turned inwards with a short posteromedial enlargement. Additionally, the hook pairs are inverted in the analyzed specimens, with the base facing upward, and they are characterized by the bulbous base, straight shaft, protruded thumb, and curved point. These morphological characteristics set the new species apart from the other congeners.

**Fig. 2:**
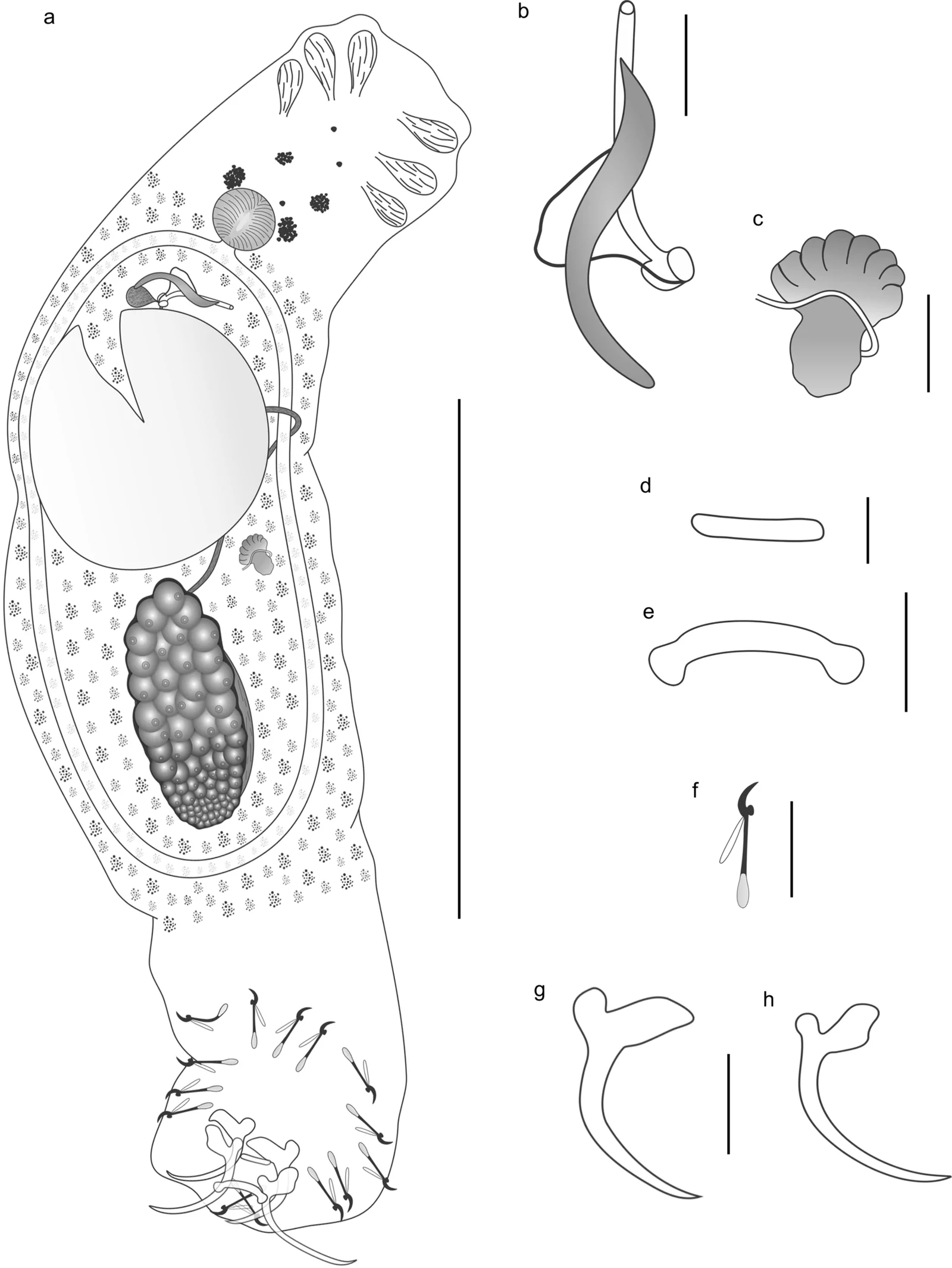
*Tocantinsiella brassicae* **n. sp.** parasite of *Caenotropus labyrinthicus* from the Tocantins River, Maranhão State, Brazil. a, Total view, ventral (composite); b, Copulatory complex, ventral view; c, Vagina, ventral view; d, Ventral bar; e, Dorsal bar; f, Hook; g, Ventral anchor; h, Dorsal anchor. Scale bars: (a) 100 μm; (b,c,f,g,h) 10 μm; (d) 5 μm; (e) 10 μm

***Tocantinsiella brassicae*** **n. sp.** Antunes, Justo, Meneses, Freitas & Cohen (Fig. 2a–h)

Type-host: *Caenotropus labyrinthicus* (Kner) (Characiformes: Chilodontidae)

Type-locality: Tocantins River, near the urban area of Imperatriz, in the village of Embiral (5°27′50″S, 47°33′48″W), Maranhão State, Brazil.

Type-material: Holotype CHIOC: 41141; Paratypes CHIOC: 41142, 41143, 41144

Site of infestation: Gills.

Parasitological indices: Prevalence 21.1% (15/71 infested); Mean abundance of infestation: 0.38; Mean intensity of infestation: 1.8; Amplitude of intensity: 1–4.

Etymology: The specific name is Latin and derived from the genus name of the cauliflowers (*brassica –* botanical genus of plants which includes cauliflower, cabbage, etc.) and refers to the shape of the vagina, which is similar to a cauliflower.

### Description (Fig. 2a–h)

(Based on 27 specimens mounted in Hoyer’s medium): Body fusiform, robust at mid-body length, 281 (170–375; n = 15) long, 84 (55–138; n = 24) wide. Tegument smooth. Cephalic lobes moderately developed, with three bilateral pairs of head organs. Two pairs of eyespots present, anterior pair smaller and closer than the posterior pair. Accessory eye granules scattered in the anterior region. Pharynx spherical, 24 (21–28; n = 9) in diameter. Two intestinal caeca confluent posterior to gonads. Germarium elongated 47 (38–55; n = 4) long, 24 (15–33; n = 4) wide. Mehlis’ gland, oviduct, ootype, and uterus not observed. Egg subspherical, 50 (n = 1) in diameter. Vaginal opening marginal, sinistral, close to the middle length of the body. Vagina robust and sclerotized, presenting a “cauliflower” shape; vaginal vestibule leaf-shaped, 16 (10–25; n = 20) long, 9 (5–12; n = 20) wide. Vitellaria present throughout the trunk, surrounding the reproductive organs. Testis dorsal to germarium. Vas deferens looping left intestinal cecum. Seminal vesicle a dilation of vas deferens, semi-oval shape. Single prostatic reservoir pouch-like shaped. Male copulatory organ (MCO) represented by a slightly sclerotized tube, 25 (16–36; n = 26) long. Accessory piece sclerotized, recurved at proximal portion, represented as a sinuous rod-shaped structure, 28 (15–36; n = 25) long. Copulatory ligament connecting the base of the MCO to the accessory piece. Haptor subspherical, 58 (45–90; n = 24) wide. Ventral bar straight, short and delicate, 10 (8–11; n = 14) wide. Dorsal bar straight with dilated slightly turned downwards, 18 (12–25; n = 13) wide. Ventral anchor delicate, with elongated superficial root and rounded deep root, long and curved shaft and curved point, 18 (12–26; n = 23) long, 11 (7–16; n = 23) wide. Dorsal anchor with well-developed grooved superficial root, narrow and rounded deep root, long and slightly curved shaft, straight point, 22 (12–31; n = 24) long, 9 (6–15; n = 24) wide. Seven pairs of hooks similar in shape and size, with equal length, 13 (11–15; n = 22) long. Hooks divided into two subunits, distal subunit corresponding ⅔ of the total shank length, slightly variable between pairs of hooks, long delicate shaft, delicate thumb and short point. Filamentous hooklet (FH) loop about 1/2 of total shank length.

Remarks: *T. brassicae ***n. sp.** is characterized by a relatively small and straight ventral bar when compared to the dorsal bar. The new species presents a simple and tubular MCO similar to *Tocantinsiella maranhensis* **n. sp.**, but differs from that species by having similar hooks in shape and size, which are divided into two subunits, and by the cauliflower-shaped morphology of the vagina.

**Fig. 3:**
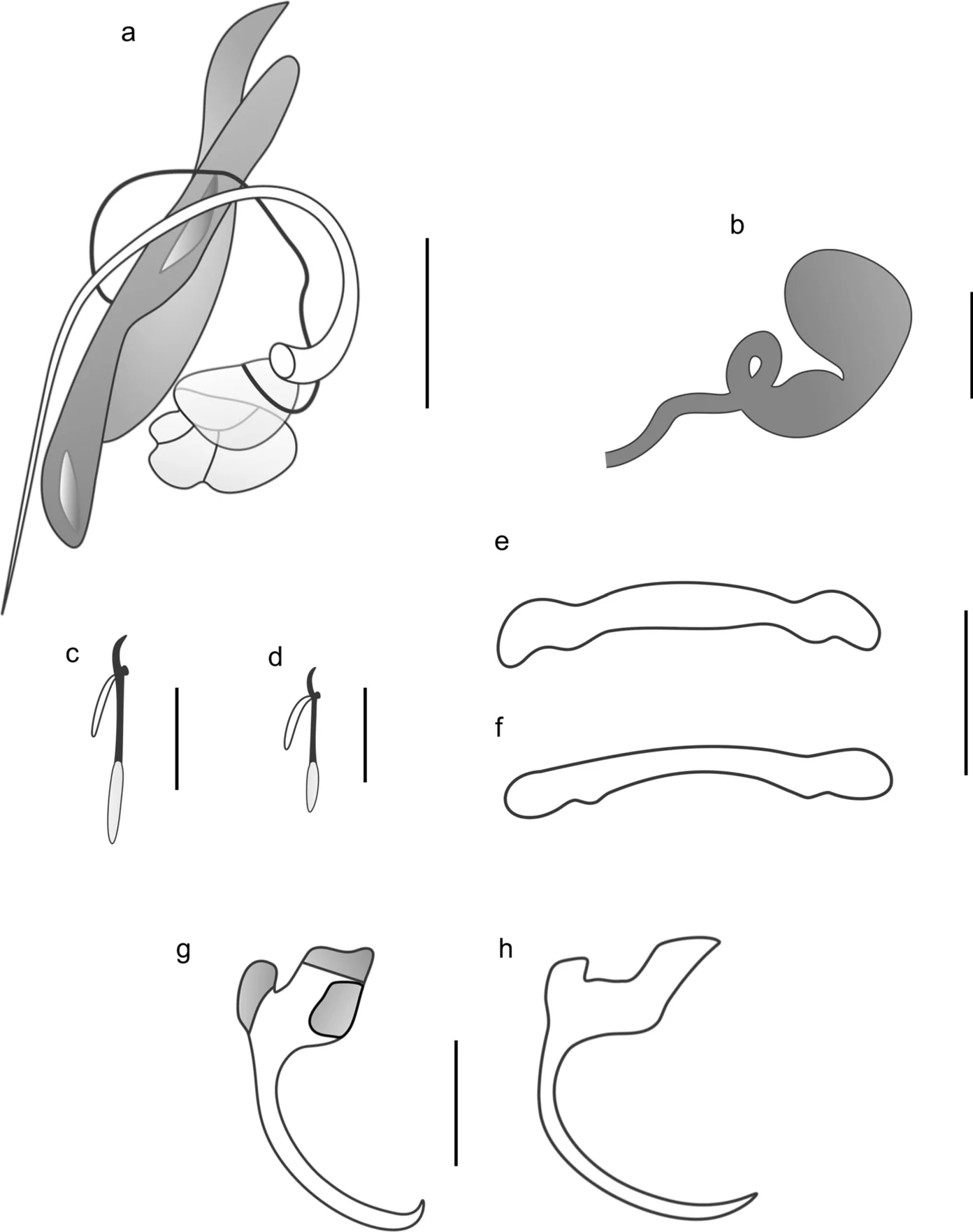
*Tocantinsiella vesiculosa* **n. sp.** parasite of *Caenotropus labyrinthicus* from the Tocantins River, Maranhão State, Brazil. a, Copulatory complex, ventral view; b, Vagina, ventral view; c, Hook pairs 2–7; d, Hook pair 1; e, Ventral bar; f, Dorsal bar; g, Ventral anchor; h, Dorsal anchor. Scale bars: (a,c,d,g,h) 10 μm; (b,e,f) 15 μm

***Tocantinsiella vesiculosa*** **n. sp.** Antunes, Justo, Meneses, Freitas & Cohen (Fig. 3 a–h)

**Fig. 4:**
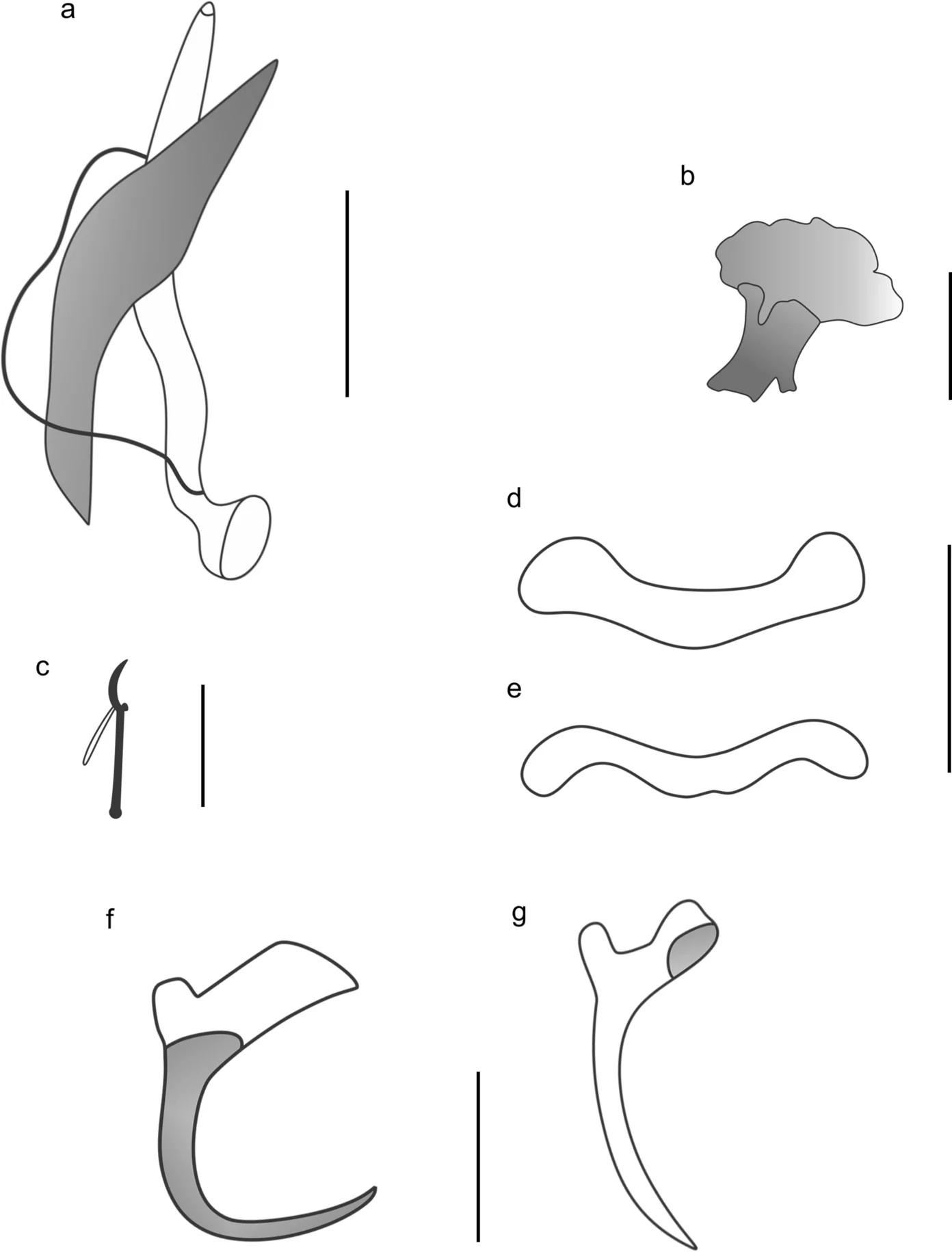
*Tocantinsiella flabellata* **n. sp.** parasite of *Caenotropus labyrinthicus* from the Tocantins River, Maranhão State, Brazil. a, Copulatory complex, ventral view; b, Vagina, ventral view; c, Hook; d, Ventral bar; e, Dorsal bar; f, Ventral anchor; g, Dorsal anchor. Scale bars: a–g 10 μm

Type-host: *Caenotropus labyrinthicus* (Kner) (Characiformes: Chilodontidae)

Type-locality: Tocantins River, near the urban area of Imperatriz, in the village of Embiral (5° 27′ 50″ S, 47° 33′ 48″ W). Maranhão State, Brazil.

Other localities: Piriá River, Gurupi River basin, Beiradão village, municipality of Paragominas (3°20′06.0″S 46°52′24.8″W). Pará State, Brazil.

Type-material: Holotype CHIOC: 41135; Paratypes CHIOC: 41136, 41137 a-b

Site of infestation: Gills.

Parasitological indices: Prevalence 4.22% (3/71 infested); Mean abundance of infestation: 0.05; Mean intensity of infestation: 1.33; Amplitude of intensity: 1–2.

Etymology: The specific name is an adjective derived from Latin (*vesiculosa* – vesiculous) and refers to the shape of the vagina, which is similar to a vesicle.

### Description (Fig. 3a–h)

(Based on 3 specimens mounted in Hoyer’s medium and one stained in Gomori’s trichrome): Body fusiform and robust, 160 and 295 (n = 2) long, 34 and 123 (n = 2) wide. Tegument smooth. Cephalic lobes moderately developed. Three bilateral pairs of head organs. Two pairs of eyespots present, anterior pair smaller and closer than posterior pair. Accessory eye granules not observed. Pharynx spherical 15 (n = 1) in diameter. Two intestinal caeca confluent posterior to gonads. Germarium elongated and oval-shaped, 13 (n = 1) long and 10 (n = 1) wide. Mehlis’ gland, oviduct, ootype, uterus and egg not observed. Vaginal opening marginal and sinistral. Vagina sclerotized and robust, with a semicircular shape, without ornamentations, reminding a bladder, 22 (15–30; n = 3) long, 14 (10–18; n = 3) wide. Vitellaria present throughout the trunk, except in the reproductive organs and copulatory complex. Testis dorsal to germarium. Vas deferens looping left intestinal cecum. Vesicle seminal a dilation of vas deferens. Single prostatic reservoir saccate. Male copulatory organ (MCO) comprising a sclerotized arcuate tube with a poorly sclerotized flanged base, 32 (27–35; n = 3) long. Accessory piece long, consisting of two straight, parallel parts of similar length that overlap, ventral part presenting two small grooves and dorsal one with a pointed tip, 29 (28–32; n = 3) long. Copulatory ligament connecting the base of the MCO to the accessory piece. Haptor subspherical, 65 (23–88; n = 3) wide. Ventral bar with a delicate groove in the central region and a slightly curved process at the ends, 35 (n = 2) long. Dorsal bar with a similar groove and curved process at the ends, 25–27 (n = 2) long. Ventral anchor with heavily sclerotized regions close to the superficial and deep roots, long superficial root, with a rectangular shape and a small cut close to the end, short and rounded deep root, long and curved shaft and curved point, 15–25 (n = 2) long, 11–16 (n = 2) wide. Dorsal anchor with superficial root developed and a rectangular shape, deep root short and rectangular at the end, elongated shaft and curved point, 15–28 (n = 2) long, 10–14 (n = 2) wide. Seven pairs of hooks similar in shape, hook pairs divided into two subunits, distal subunit corresponding almost 1/2 of the total shank length, long shaft, protruding thumb, and short point, 20 (15–23; n = 5) long; hook pair 1 smaller than the others with a length of 15μm. Filamentous hooklet (FH) loop about 1/3 of total shank length.

Remarks: *Tocantinsiella vesiculosa* **n. sp.** are distinguished from other species within the genus due to the presence of a poorly sclerotized flanged base on its MCO and an accessory piece consisting of two straight parts that overlap themselves. In addition, the vagina is dilated and bladder shaped. The new species presents hook pairs with two subunits similar to *T. brassicae* **n. sp.**; however, in *T. vesiculosa* **n. sp.**, hook pair 1 is smaller than the other hook pairs. The new species can also be distinguished from its congeners by the pronounced sclerotization of the ventral anchor.

***Tocantinsiella flabellata* n. sp.** Antunes, Justo, Meneses, Freitas & Cohen (Fig. 4a–g)

Type-host: *Caenotropus labyrinthicus* (Kner) (Characiformes: Chilodontidae)

Type-locality: Tocantins River, near the urban area of Imperatriz, in the village of Embiral (5° 27′ 50″ S, 47° 33′ 48″ W). Maranhão State, Brazil.

Type-material: Holotype CHIOC: 41145 a; Paratypes CHIOC: 41145 b-c, 41146

Site of infestation: Gills.

Parasitological indices: Prevalence 5.63% (4/71 infested); Mean abundance of infestation: 0.09; Mean intensity of infestation: 1.75; Amplitude of intensity: 1–3.

Etymology: The specific name is an adjective derived from Latin (*flabellata* – fan-shaped) and refers to the shape of the vagina, which is similar to a fan.

### Description (Fig. 4a–g)

(Based on 7 specimens mounted in Hoyer’s medium): Body fusiform, 288 (255–320; n = 2) long, 76 (68–83; n = 4) wide. Tegument smooth. Cephalic lobe slightly developed. Three pairs of head organs. Two pairs of eyespots present, anterior pair smaller and closer than posterior pair. Accessory eye granules absent. Pharynx spherical, 23 (16–30; n = 3) diameter. Two intestinal caeca confluent posterior to gonads. Germarium, oviduct, Mehlis gland, ootype, uterus, egg, and testis not observed. Vaginal opening marginal, sinistral, at the middle region of the body. Vagina sclerotized, robust, presenting in the shape of a “fan”, 13 (10–15; n = 3) long, 10 (6–13; n = 3) wide. Vitellaria present throughout the trunk, except for the reproductive organs’ areas and the copulatory complex. Vas deferens looping left intestinal cecum. Seminal vesicle a dilation of vas deferens, in semi-oval shape. Single prostatic reservoir pouch-like shaped. Male copulatory organ (MCO) comprising a simple long tube, 23 (20–30; n = 7) long. Accessory piece elongated and sclerotized, widening at midlength, 21 (16–28; n = 7) long. Copulatory ligament connecting the base of the MCO to the accessory piece. Haptor subspherical, 48 (30–58; n = 5) wide. Ventral bar slightly arched with rounded ends, 15 (14–16; n = 5) long. Dorsal bar elongated, with curved ends and a delicate groove in the median region, 15 (8–18; n = 6) long. Ventral anchor with elongated superficial root, rectangular in shape at the end, deep root short and moderately rounded, shaft more sclerotized than the superficial and deep root regions and curved point, 18 (12–23; n = 6) long, 13 (11–16; n = 6) wide. Dorsal anchor with superficial root elongated and rectangular, with heavily sclerotized region, deep root short and delicate, long shaft and slightly curved point, 19 (17–20; n = 6) long, 7 (3–11; n = 6) wide. Seven pairs of hooks similar in shape and size, with delicate and long shank, protruding thumb, slightly curved shaft, curved point and bulbous base, 13 (11–14; n = 6) long. Filamentous hooklet (FH) loop about 1/2 of the total shank length.

Remarks: *Tocantinsiella flabellata ***n. sp.** closely resembles *Tocantinsiella maranhensis* **n. sp.** in terms of the morphology of its hooks, which have a bulbous appearance at the base. However, it differs in the position of the hooks, which are inverted in *T. maranhensis* **n. sp*.*** The new species can also be distinguished from other species described herein by the presence of a ventral anchor with a visible sclerotization that extends along the length of the shaft, as well as ornamented regions present in the dorsal anchor. In addition, *T. flabellata* **n. sp*.*** has a robust and sclerotized vaginal vestibule, presenting in the shape of a “fan.”

## Discussion

The diversity of parasitic fauna in Chilodontidae hosts remains poorly understood, with records of only two genera of Monopisthocotyla parasitizing fishes of this family so far: *Anacanthorus* Mizelle & Price, 1965 and *Urocleidoides* Mizelle & Price, 1964. This study proposed the description of *Tocantinsiella* **n. gen.** based on morphological data and described four species of Dactylogyridae parasitizing *C. labyrinthicus* collected from the Tocantins River. The findings expand the known diversity of the host’s parasitic fauna and provide consistent morphological evidence supporting the proposal of a new genus and four new species.

*Tocantinsiella ***n. gen.** was proposed to accommodate species that present a sclerotized, robust vaginal vestibule in a sinistral position, and a copulatory ligament connecting the accessory piece to the male copulatory organ. The most closely related genus within Dactylogyridae that resembles the proposed new genus is *Philocorydoras*. However, species of *Philocorydoras* have been described from the gills of siluriform fishes, a distinct host group. The morphological characteristics present in *Tocantinsiella* **n. gen.** allowed to propose a new genus and allocate four newly described species. Further molecular analyses would provide data to study the phylogenetic interrelationships among species of the new genus and other species of Dactylogyridae, as done by Acosta et al. (2019) for species of *Nanayella* Acosta, Mendoza-Palmero, Silva & Scholz, 2019 and to determine whether *Tocantinsiella* **n. gen.** is phylogenetically related to *Philocorydoras*.

Until the present study, there have been four reports of Monopisthocotyla in *C. labyrinthicus*: the description of two new species and new data of *Urocleidoides* spp. and new data for *Anacanthorus simpliciphallus* (Freitas et al., 2021, 2025; Bezerra et al., 2023). The proposal of *Tocantinsiella ***n. gen.** and the description of four new species highlight the diversity and richness of the helminth fauna present in the host studied and draws attention to the importance of expanding research related to certain groups of helminth parasites of freshwater fishes that still require further studies, including in the family Chilodontidae.

Interestingly, Chilodontidae is closely related to other characiform families that typically host species of *Anacanthorus* and *Urocleidoides*. This may suggest that the dispersal and diversification of these monopisthocotylans among Neotropical characiforms may be constrained by host phylogenetic proximity. Braga et al. (2015) pointed out that cospeciation events among Monopisthocotyla are also explained by events of host-switching or non-vicariant modes. In addition, these authors suggested that the phylogenetic contiguity observed within Characiformes allowed parasites to disperse to distant hosts while simultaneously colonizing phylogenetically related groups, a mechanism termed ‘stepping-stone’ host-switching. In this context, it would not be surprising if further studies on the diversity of Monopisthocotyla parasites of Characiformes reveal species of *Tocantinsiella* **n. gen.** in other host families.

## Data Availability

No datasets were generated or analysed during the current study.
